# Beliefs about Appropriate Antibacterial Therapy, California

**DOI:** 10.3201/eid1107.050112

**Published:** 2005-07

**Authors:** Kate C. Cummings, Jon Rosenberg, Duc J. Vugia

**Affiliations:** * California Department of Health Services, Berkeley, California, USA

**Keywords:** Antibiotic resistance, attitudes, beliefs, antibiotics, minorities, women

## Abstract

To our knowledge, previous population-based surveys have not assessed misconceptions about antibacterial drug use over time. We documented a 26.3% decline in a key misconception in California women in 2003 compared to 2000; declines varied significantly by education level. Educational campaigns specifically designed to influence important subpopulations are needed.

Antibacterial drug–resistant bacteria pose a substantial challenge to public health. Inappropriate use of antibacterial drugs, such as using them to treat viral respiratory infections, accelerates the emergence of resistant organisms (1,2). Nonclinical factors, such as patient expectation and demand, contribute to inappropriate antibacterial prescribing (3–6). To address patient misconceptions and physician prescribing behavior, the Centers for Disease Control and Prevention, along with states and private health organizations, initiated educational programs and campaigns to encourage the judicious use of antimicrobial drugs (7). In 1999, the California Medical Association Foundation initiated the Alliance Working for Antibiotic Resistance Education (AWARE) project. AWARE, a coalition of public and private organizations, was launched to educate physicians and consumers about appropriate antibacterial drug use. In 2003, AWARE worked with a coalition of minority physicians, including Asian physicians, to reduce inappropriate antibacterial use in minority communities (Elissa Maas, pers. comm.).

To date, few studies assess the effect of these educational campaigns over time, especially among underserved populations. We surveyed a population-based sample of California women to identify the magnitude of and characteristics independently associated with the misconception that a cold or flu should usually be treated with an antibacterial drug in 2000 and 2003. We assessed temporal trends in the prevalence of this misconception by comparing the prevalence reported in 2003 with that reported in 2000 for the entire study sample and by specific risk characteristics.

## The Study

The California Women's Health Survey is an ongoing, monthly telephone survey that collects information on health-related behavior and attitudes from randomly selected adult women (8,9). Trained staff interviewed participants in 2000 (n = 4,012) and 2003 (n = 4,004) in English (n = 7,058) or Spanish (n = 958). Participants reported race, ethnicity, country of birth, year of entrance into the United States, and current age, county of residence, number and age(s) of children, educational attainment, household income, and access to healthcare coverage. We defined the key misconception as an affirmative answer to the following dichotomized question: "Antibiotics, such as penicillin, doxycycline, or amoxicillin, are used to treat a variety of medical conditions; do you believe that a cold or flu should usually be treated with antibiotics?"

We calculated the crude proportion of women who held this misconception by year with weighted proportions and Mantel-Haenszel chi-square statistics that adjusted each sample to the age and race distribution of the 1990 California population (8,9). We examined the relationship between the misconception and the characteristics listed in Table 1 by year of study by using prevalence proportions and unconditional logistic regression. To produce comparable results, multivariate models for 2000 and 2003 included all variables that demonstrated a significant association with the misconception in either year in univariate analyses. We developed a third multivariate model that combined data from both years to assess the independent association between the misconception and year of study. While we used logistic regression to assess for confounding, we present unadjusted prevalence ratios and calculate relative prevalence reductions to measure changes in the misconception over time. We did so because the unadjusted and adjusted results by logistic regression were equivalent (which suggests an absence of confounding) and because the odds ratio did not reasonably approximate the prevalence ratio for women without a high school education (10). We considered p values ≤0.01 to be significant, but we present 95% confidence intervals (CIs) to facilitate comparing our results to those from other studies. We restricted all models to participants who gave usable responses to all variables (n = 7,430) and assessed models for potential 2-way interactions, goodness of fit, and potential colinearity.

**Table 1:** Factors associated with the misconception that cold or flu should be treated with antibacterial drugs, California, 2000 and 2003*

Characteristic	% in 2000† (n = 3,703) (OR‡, 95% CI)	% in 2003† (n = 3,727) (OR‡, 95% CI)
Race/ethnicity		
Black, non-Hispanic	40.6 (2.57, 1.88–3.52)	32.5 (2.82, 1.98–4.08)
Hispanic	46.2 (2.00, 1.58–2.52)	39.5 (1.96, 1.51–2.53)
Asian/other	37.1 (2.67, 1.94–3.67)	19.7 (1.78, 1.21–2.62)
White, non-Hispanic	19.4 (referent)	13.8 (referent)
Age (y)		
18–34	36.1 (2.13, 1.74–2.61)	26.7 (1.55, 1.23–1.94)
35–54	18.9 (referent)	14.5 (referent)
≥55	28.5 (1.59, 1.27–1.97)	19.7 (1.24, 0.97–1.58)
Annual household income ($US)		
≤14,999	46.9 (2.48, 1.91–3.22)	41.9 (3.10, 2.34–4.10)
15,000–24,999	36.8 (2.03, 1.56–2.64)	29.3 (1.94, 1.44–2.62)
25,000–49,999	25.9 (1.62, 1.30–2.03)	20.3 (1.70, 1.32–2.19)
≥50,000	12.9 (referent)	8.5 (referent)
Education		
<12 y	52.2 (3.03, 2.23–4.10)	43.1 (3.16, 2.26–4.44)
High school graduate/GED	31.8 (1.91, 1.50–2.44)	29.3 (3.08, 2.34–4.07)
Some college	20.5 (1.33, 1.04–1.69)	15.7 (1.79, 1.35–2.36)
College graduate	13.9 (referent)	7.3 (referent)
Region of California§		
Central	28.2 (1.34, 1.04–1.74)	24.8 (1.65, 1.25–2.17)
Southern	29.9 (1.40, 1.16–1.70)	21.4 (1.38, 1.10–1.71)
Northern	18.7 (referent)	13.1 (referent)
Years in United States		
≤5	57.1 (2.03, 0.86–4.79)	42.7 (1.58, 0.97–2.58)
6–10	50.8 (1.52, 0.98–2.35)	34.6 (1.24, 0.79–1.94)
>10	36.6 (0.98, 0.77–1.23)	29.6 (1.11, 0.85–1.44)
US-born	21.9 (referent)	15.3 (referent)
Children <6 y in household		
Yes	28.9 (0.61, 0.49–0.76)	24.9 (0.86, 0.68–1.09)
No	25.0 (referent)	17.5 (referent)
Access to health plan		
No	44.1 (1.06, 0.84–1.35)	37.2 (1.30, 1.02–1.65)
Yes	22.9 (referent)	16.4 (referent)

*OR, odds ratio; CI, confidence interval; GED, general equivalency diploma. †Percent represents number of respondents with misconception divided by total number of respondents. Percentages for race/ethnicity and age are weighted to the 1990 California population; all other percentages are unweighted. ‡Adjusted for all characteristics listed in the table. §Central = Fresno, Kern, Kings, Madera, Merced, Monterey, San Joaquin, San Luis Obispo, Santa Barbara, Santa Cruz, Stanislaus, and Tulare Counties; southern = Imperial, Los Angeles, Orange, Riverside, San Bernardino, San Diego, and Ventura counties; northern = all remaining Counties.

In California, 21.0% of women surveyed in 2003 believed a cold or flu should usually be treated with an antibacterial drug. Table 1 lists factors independently associated with this key misconception among California women. In both 2000 and 2003, the unadjusted prevalence of the misconception was greatest among women who were Hispanic, 18–34 years of age, had an annual household income ≤$14,999, had <12 years of education, resided in central or southern California, had lived in the United States for ≤5 years, had children <6 years of age in the household, or had no access to a healthcare plan.

We observed a 26.3% decline in the prevalence of the misconception in all women surveyed in 2003 compared with those surveyed in 2000 (21.0% vs. 28.5%, p<0.001). Table 1 shows consistent declines across risk characteristics, with several notable exceptions. First, we detected a strong statistical interaction between education and year (p = 0.007); women with a college diploma were 47.0% less likely and women without a high school diploma were 17.0% less likely to report the misconception in 2003 than those surveyed in 2000 (Table 2). Women with a high school diploma were as likely to report the misconception in 2000 and 2003. Second, although not significant, after adjusting for other associated factors, Asian women were 55.7% less likely to report the misconception in 2003 compared with those surveyed in 2000. Other women were, on average, 24.0% less likely to report the misconception in 2003 than in 2000. Among women ≥55 years of age, African American women were as likely to report the misconception in 2003 as 2000, and their adjusted odds ratio for the misconception increased from 3.1 (95% CI 1.70–5.67) in 2000 to 5.3 (95% CI 2.67–10.52) in 2003 (detailed data on race stratified by age and year not shown).

**Table 2:** Decline in the misconception, by education level, that cold or flu should be treated with antibacterial drugs, California, 2000 and 2003*

Education level	%† (PR, 95% CI)	% decrease‡
<12 y		
2003 study	43.1 (0.83, 0.72–0.95)	17.0
2000 study	52.2 (referent)	
High school graduate/GED		
2003	29.3 (0.92, 0.80–1.06)	8.0
2000	31.8 (referent)	
Some college		
2003	15.7 (0.77, 0.64–0.92)	23.0
2000	20.5 (referent)	
College graduate		
2003	7.3 (0.53, 0.42–0.67)	47.0
2000	13.9 (referent)	

*PR, unadjusted prevalence ratio; CI, confidence interval, GED, general equivalency diploma. †Number respondents with misconception divided by total number respondents. ‡Percent decrease from 2000 to 2003.

## Conclusions

Our survey results show a 26.3% decline from 2000 to 2003 in the misconception among California women that a cold or flu should usually be treated with an antibacterial drug. This finding suggests that general educational campaigns before and during this period may have contributed to moderate reductions in the prevalence of misconceptions about antibacterial drug use. Although this study was not designed to evaluate specific interventions, our study period and population-based sample can provide an ecologic assessment of changing beliefs.

The decline in the misconception was greatest among women who were non-Hispanic white or Asian, had at least some college education, or had higher household incomes. Among levels of education, why women who graduated from high school had the smallest decline is unclear. This finding may reflect a true unexplained finding, an artifact of the sample (selection or information bias), or chance. The inverse relationship between the misconception and the presence of children <6 years of age in the household in 2000 may indicate success in initial educational campaigns focused on pediatricians and mothers with young children; that this relationship becomes less significant in 2003 may indicate that other women are "catching up."

Our findings, drawn from a large, ethnically diverse, population-based sample, show that 21.0% of California women in 2003 still held the misconception, particularly women who were younger, black, or Hispanic; had lower educational attainment or household income; resided in central and southern California; or had no healthcare coverage. Although not restricted to women, other studies identified similar demographic and socioeconomic risk groups (11,12). Social services intended to reach persons with these characteristics may be important venues for education on appropriate antibacterial drug use.

Cultural and social perspectives of disease, including differences in antibacterial drug regulations that affect the availability and cost of drugs in immigrants' countries of birth, can play an important role in misconceptions about antibacterial drugs (13,14). We documented racial and ethnic variation in California women with the misconception, despite adjusting for selected socioeconomic factors; the remaining variation likely reflects unmeasured socioeconomic or cultural factors. We note, in particular, the persistent prevalence of the misconception in African American women and the decreased prevalence of it in Asian women. While this study could not evaluate specific interventions, some California AWARE activities were focused on Asian physicians during 2003. Other research has documented racial and ethnic variation in parental expectations of antibacterial drugs (15). New intervention studies and campaigns aimed at educating and influencing women in specific minority, education, and income subgroups about appropriate antibacterial drug use are needed.

Our study has several limitations. First, although women may be more likely to be the health decisionmakers for young children and for the household, we do not know if our results can be generalized to men. Second, we did not have sufficient sample size to explore specific subgroups within the Asian race category. Third, we only included women in households with telephones who spoke either Spanish or English and chose to participate. Fourth, we were only able to interview women in Spanish or English, which may limit comparability between immigrant Hispanic and Asian women. Finally, this misconception may not equivocate to antibacterial drug expectation or demand.

In conclusion, our surveys document a decline in a key misconception in California women about appropriate antibacterial drug use and support continuation of educational campaigns to address this public health problem. Further refining and focusing of these educational efforts are needed to reach minority women and women with lower educational attainment and lower household incomes.

## References

[1] Kunin CM. Resistance to antimicrobial drugs: a worldwide calamity. Ann Intern Med. 1993;118:557–61. 8442626 10.7326/0003-4819-118-7-199304010-00011

[2] Neu HC. The crisis in antibiotic resistance. Science. 1992;257:1064–73. 10.1126/science.257.5073.1064 1509257

[3] MacFarlane J, Holmes W, MacFarlane R, Britten N. Influence of patients' expectations on antibiotic management of acute lower respiratory tract illness in general practice: questionnaire study. BMJ. 1997;315:1211–4. 10.1136/bmj.315.7117.1211 9393228 PMC2127752

[4] Barden L, Dowell S, Schwartz B, Lackey C. Current attitudes regarding use of antimicrobial agents: results from physicians and parents' focus group discussions. Clin Pediatr (Phila). 1998;37:665–72. 10.1177/000992289803701104 9825210

[5] Mangione-Smith R, McGlynn EA, Elliott MN, Krogstad P, Brook RH. The relationship between perceived parental expectations and pediatric antimicrobial prescribing behavior. Pediatrics. 1999;103:711–8. 10.1542/peds.103.4.711 10103291

[6] Dosh SA, Hickner JM, Mainous AGI, Ebell MH. Predictors of antibiotic prescribing for nonspecific upper respiratory infections, acute bronchitis, and acute sinusitis; a UPRNet study. J Fam Pract. 2000;49:407–13. 10836770

[7] Centers for Disease Control and Prevention. Preventing emerging infectious diseases; addressing the problem of antimicrobial resistance: a strategy for the 21st Century. [cited 2005 Jan 23]. Available from http://www.cdc.gov/ncidod/emergplan/antiresist/antimicrobial.pdf

[8] California Department of Health Services and the Public Health Institute. California Women's Health Survey. SAS dataset documentation and technical report. 2000.

[9] California Department of Health Services and the Public Health Institute. California Women's Health Survey. SAS dataset documentation and technical report. 2003.

[10] Deeks J. When can odds ratios mislead? Odds ratios should be used only in case-control studies and logistic regression analyses [letter]. BMJ. 1998;317:1155–6. 10.1136/bmj.317.7166.1155a 9784470 PMC1114127

[11] Eng JV, Marcus R, Hadler JL, Imhoff B, Vugia DJ, Cieslak PR, Consumer attitudes and use of antibiotics. Emerg Infect Dis. 2003;9:1128–33. 14519251 10.3201/eid0909.020591 PMC3016767

[12] Corbett KK, Gonzales R, Leeman-Castillo BA, Flores E, Maselli J, Kafadar K. Appropriate antibiotic use: variation in knowledge and awareness by Hispanic ethnicity and language. Prev Med. 2005;40:162–9. 10.1016/j.ypmed.2004.05.016 15533525

[13] Harbarth S, Albrich W, Brun-Buisson C. Outpatient antibiotic use and prevalence of antibiotic-resistance pneumococci in France and Germany: a sociocultural perspective. Emerg Infect Dis. 2002;8:1460–7. 12498664 10.3201/eid0812.010533 PMC2738507

[14] McKee MD, Mills L, Mainous AG III. Antibiotic use for the treatment of upper respiratory infections in a diverse community. J Fam Pract. 1999;48:993–6. 10628580

[15] Mangione-Smith R, Elliott MN, Stivers T, McDonald L, Heritage J, McGlynn EA. Racial/ethnic variation in parent expectations for antibiotics: implications for public health campaigns. Pediatrics. 2004;113:385–94. 10.1542/peds.113.5.e385 15121979

